# The Health Ministry's Record

**Published:** 1921-02-19

**Authors:** 


**THE PUBLIC WELL-BEING**—(*continued*).

### The Health Ministry's Record.

### SIR KINGSLEY WOOD'S DEFENCE.

The series of articles which Sir Kingsley Wood, M.P., Parliamentary Secretary to the Health Minister, has been contributing to the *Daily Telegraph* constitutes an interesting review of the new Ministry's work. It is of real value also to the social reformer, because it puts the facts clearly and with moderation. One may see from this broad account of the activities of Dr. Addison's Department the immense scope of its work and responsibilities, and the complicated and heavy problems which it has had and still has to face. The Ministry has many critics, some honest and well-meaning, others for political or other reasons unscrupulous and vicious; but on balance it would be ungenerous to withhold recognition of the resolute way in which, under Dr. Addison, the new public-health administration has tackled its manifold difficulties. In the past the various criticisms have been along two separate channels. The first contained the view of those who considered that the Ministry was doing too much and infringing on the rights of individuals; the second contained the view of those who accused the Department of bungling incompetence in everything which it undertook. Now the criticisms have taken a new turn. The argument appears to be that, having regard to the financial situation and the need for economy, the first Department in which a drastic cutting-down policy must be adopted is that which ministers to the health of the people, with the Board of Education a good second.

Sir Kingsley Wood's articles are obviously designed to show not only that the measures carried into effect by the Ministry have been at a relatively low cost financially, but that so-called economy in matters of public health, apart from the condition of giving the nation good value for its money, is the falsest economy of all. With regard to the first point, he asks us to note that while rates generally in all boroughs have risen enormously since the war, the maternity and child-welfare services in most places are costing less than a penny rate. The same is true of the municipal provision for the treatment of tuberculosis; and the treatment for venereal disease is usually met by a rate of about a farthing in the pound. Very little increase in the rates is, in fact, due to the direct cost of health administration and social reforms. It is perfectly true that there is no balance sheet which requires more careful attention and scrutiny than the National Health balance sheet. What, Sir Kingsley Wood pertinently asks, will be the cost to the State of the million defective children in our schools to-day? How many millions sterling have we to pay on account of the million persons living in slum areas? Tuberculosis is as prevalent as smallpox was in the seventeenth century. How much money is that costing the nation? We are hearing a good deal about waste, and much of this angry talk may be justified. But it is a waste and extravagance to permit such conditions to continue, and the nation is paying, and will pay, a long bill for it. A large measure of the industrial unrest from which we are now suffering, damaging to trade and business in every form, is due to the existence of preventable disease and bad health conditions in large elements of the community.

As regards general health work, it is claimed that through the Nurses' Registration Act the Ministry has already done much to secure an improved standard of nursing. The Dentists' Registration Bill, now presented by Dr. Addison to Parliament, will, in similar fashion, Sir Kingsley Wood thinks, do much to regularise and classify the practice of dentistry, while the Proprietary Medicines Bill will be useful in dealing with abuses affecting the sale of certain types of patent medicines. There has been a valuable extension of maternity and child-welfare work. Centres for ante-natal and infant-welfare work are rapidly increasing, and many local authorities are employing women doctors specially for the work. Efficient midwifery services in rural areas are being developed, and it should be noted that the infantile mortality in 1919—89 per 1,000—was the lowest on record. "We have got to have a great sanitary cordon encircling round our whole coast," adds the defender of the Ministry, "as a

**THE PUBLIC WELL-BEING**—(*continued*).

barrier against the introduction of disease from abroad; and increased assistance is being given to the port sanitary authorities." The Ministry is also doing much useful work under the provisions of the Blind Persons Act; annual grants are made to ninety-four voluntary institutions, and all such agencies are carefully inspected. We have no space to deal with Sir Kingsley Wood's defence of the housing policy of the Ministry. That is a subject we may return to later. But, while we are disinclined to hold the Government blameless for all the delays and difficulties that have arisen, a good case is certainly made out for the claim that, in spite of everything, the housing question has been handled with more foresight and boldness in Great Britain than in any other country where the problem is equally acute. On the whole, the Ministry of Health can show a good record of service during its short term of existence, and its plans hold out infinite promise for the future. What is quite certain is that we cannot afford to starve it of money or strangle it with obstruction. We agree entirely with the dictum that "bad health breeds Bolshevism, and the healthy citizen stands for sanity and security."

